# B-chronic lymphocytic leukemia showed triple transformation, to diffuse large B cell, CD20-negative, and T-cell neoplasm during ofatumumab treatment: a case report

**DOI:** 10.1186/s12907-018-0072-5

**Published:** 2018-05-22

**Authors:** Osamu Imataki, Makiko Uemura

**Affiliations:** Division of Hematology, Department of Internal Medicine, Faculty of Medicine, Kagawa University, 1750-1 Ikenobe, Miki-town, Kita-county, Kagawa, 761-0793 Japan

**Keywords:** Chronic lymphocytic leukemia (CLL), Ofatumumab, Transformation, Richter’s syndrome

## Abstract

**Background:**

Chronic lymphocytic leukemia (CLL) is a mature lymphoid neoplasm currently categorized as an indolent type of malignant lymphoma. CLL progresses slowly over years, but it eventually transforms to a more aggressive lymphoma such as the diffuse large B-cell (DLBCL) type, also known as Richter’s syndrome.

**Case presentation:**

We treated a 69-year-old Japanese male who was histologically diagnosed with Richter’s syndrome after 6 years of CLL. His lymphadenopathy had systemically progressed for years, with lymphocyte counts of less than 10,000 cells/μL and a disease status of Rai classification stage I and Binet classification B. He had high fever and hepatosplenomegaly upon Richter’s transformation. The patient was treated with ofatumumab for refractory CLL, which relieved his febrile lymphadenopathy. He received a total of 11 ofatumumab courses and achieved partial remission. On the day of the 12th course of ofatumumab, his disease relapsed with febrile lymphadenopathy. Computed tomography revealed multiple liver masses and systemic lymphadenopathy, while a liver biopsy confirmed T-cell lymphoma. Concomitantly, CD20-lacking CLL cells were detected in his peripheral blood and bone marrow, and pathological examination of his left cervical lymph node biopsy showed CD20-positive DLBCL. The final diagnosis was three different types of lymphoma pathologies: (1) CD20-positive DLBCL of the lymph nodes, (2) CD20-lacking CLL of the peripheral blood and bone marrow, and (3) peripheral T-cell lymphoma (PTCL) of the liver. He received intravenous and oral dexamethasone therapy as palliative care. He died because of the rapid progression of abdominal masses 2 months after the diagnosis of triple transformation CLL. An autopsy revealed aggressive PTCL with aggressive systemic involvement of the liver, spleen, gall bladder, pericardium, bone marrow, and mediastinal–paraaortic–intraceliac lymph nodes. T-cell receptor study of an autopsy specimen supported the diagnosis of PTCL that spread to the intraceliac organs and lymph nodes. We concluded that his pathogenicity progressed to a mixture of triple lymphoma as a result of double malignant transformations, which included PTCL from CLL, CD20-negative CLL, and CD20-positive DLBCL by Richter’s transformation.

**Conclusions:**

Our case provides information on the biology of CLL, to transform from a low-grade chemosensitive status to a malignant chemoresistant status.

## Background

Richter’s syndrome is defined as the development of high-grade lymphoma in patients with chronic lymphocytic leukemia (CLL), previously diagnosed as small lymphocytic lymphoma [1, 2]. Occasionally, transformation to other types of hematological malignancies, including high-grade prolymphocytic leukemia or acute leukemia, can occur. This concept includes various possibilities for malignant transformation to more aggressive types of lymphoid malignancies. In some rare cases, the cell lineage could even be altered; for instance, from B-cell neoplasm to T-cell neoplasm. Although there have been numerous reports describing the transformation of B-CLL to diffuse large B-cell lymphoma (DLBCL), the transformation to T-CLL and T-prolymphocytic leukemia has been rarely reported [3, 4]. Thus, the various patterns of transformation are referred to as Richter’s syndrome in the literature. We encountered a case of transformation of B-CLL to DLBCL followed by peripheral T-cell lymphoma (PTCL).

Ofatumumab is an anti-CD20 antibody indicated for refractory/relapsed CLL that has been commercially available in Japan since April 2012. On October 27, 2009, the Food and Drug Administration (FDA) approved ofatumumab (Arzerra®, GlaxoSmithKline) for treatment of CLL. CLL is recognized as a slowly progressive hematological disease [5], with a number of mature lymphocytic tumor cells proliferating or circulating in the bone marrow or peripheral blood. On the other hand, Richter’s syndrome is described as an aggressive hematological malignancy that occurs during the histological transformation of CLL. Given that Richter’s syndrome exerts a progressive and aggressive clinical course within a couple of weeks, treatment should be initiated as soon as possible [1–3]. However, the efficacy of ofatumumab for treatment of Richter’s syndrome is still under investigation. The present case provides information on the biology of CLL, which has a tendency to transform from a low-grade chemosensitive status.

## Case presentation

The patient was a 64-year-old male who was diagnosed with CLL 6 years and 5 months before presentation. The patient had no family history of cancer/lymphoma and was not considered to be at a higher risk of cancer due to smoking. His blood data on admission was as follows: white blood cell count of 11,070/μL, hemoglobin concentration of 15.0 g/dL, and platelet count of 15.8 × 10^4^/μL. A physical examination showed systemic lymphadenopathy of the cervical, axillary, and inguinal regions. He sometimes received oral cyclophosphamide and prednisolone for better control of lymphocytosis (lymphocyte count > 10,000 cells/μL). His lymphadenopathy systemically progressed over the course of several years and mainly included bilateral cervical, intra-abdominal, paraaortic, and bilateral femoral lesions. Nevertheless, he remained treatment-free with a clinical status of Rai classification stage I and Binet classification B. He was treated with fludarabine-based chemotherapy for his febrile status, which was occasionally complicated. Upon reaching the age of 69 years, his CLL transformed into aggressive lymphoma, which was histologically diagnosed as DLBCL in his cervical lymph node. His clinical presentation during the transformation revealed leukocytosis, hepatosplenomegaly, and high fever with chills.

**Fig. 1:**
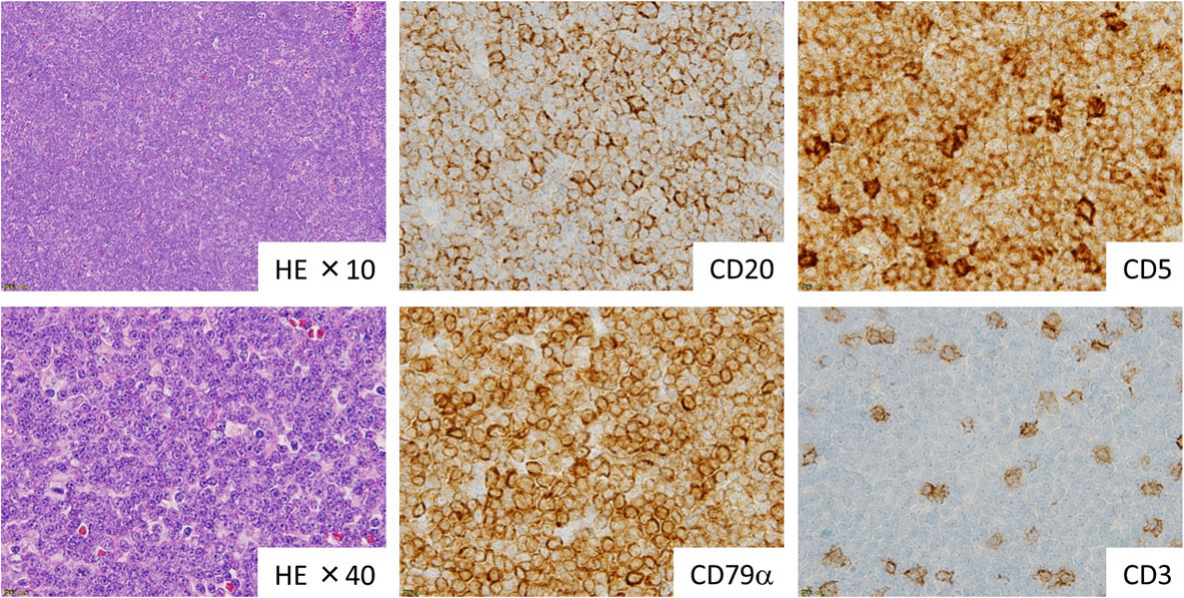
Pathology of CD20-positive diffuse large B-cell lymphoma from the lymph nodes biopsy. EBV was negative. Cervical lymph node biopsy showed diffuse growth of mid-sized lymphocytes. Immunohistochemical analysis revealed proliferating lymphoma cells that were positive for CD20, CD79α, and CD5, but negative for CD3

**Fig. 2:**
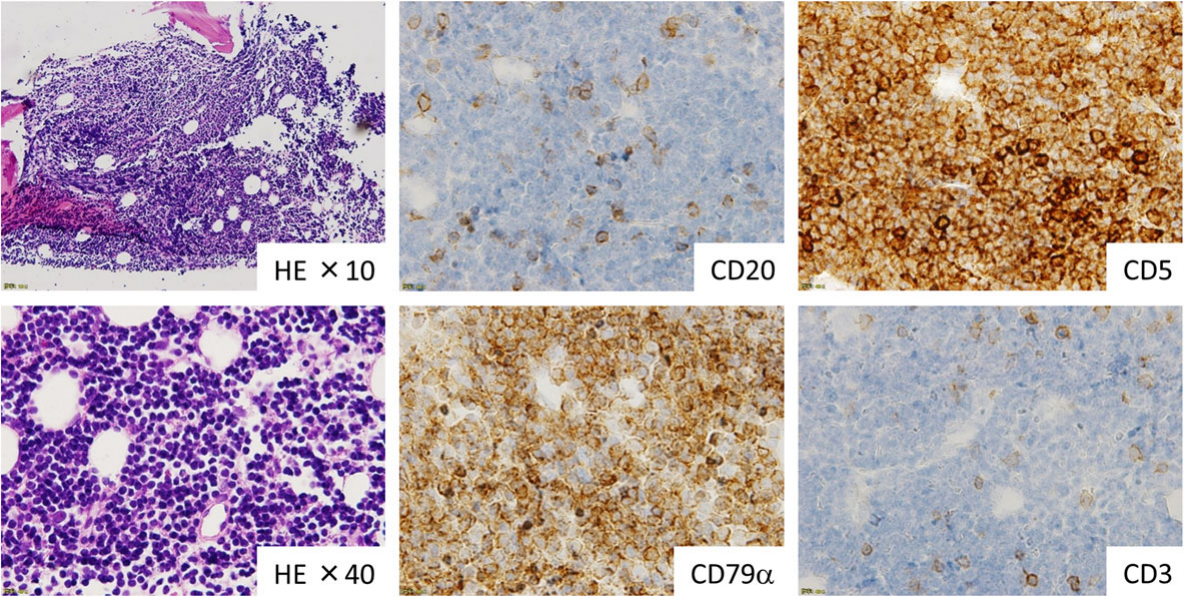
Pathology of CD20-lacking chronic lymphocytic leukemia in the bone marrow biopsy. Bone marrow biopsy revealed hypercellular marrow with diffuse proliferation of small-sized lymphocytes. Immunohistochemical analysis revealed that the proliferating lymphoma cells were positive for CD79α and CD5, but negative for CD20

**Fig. 3:**
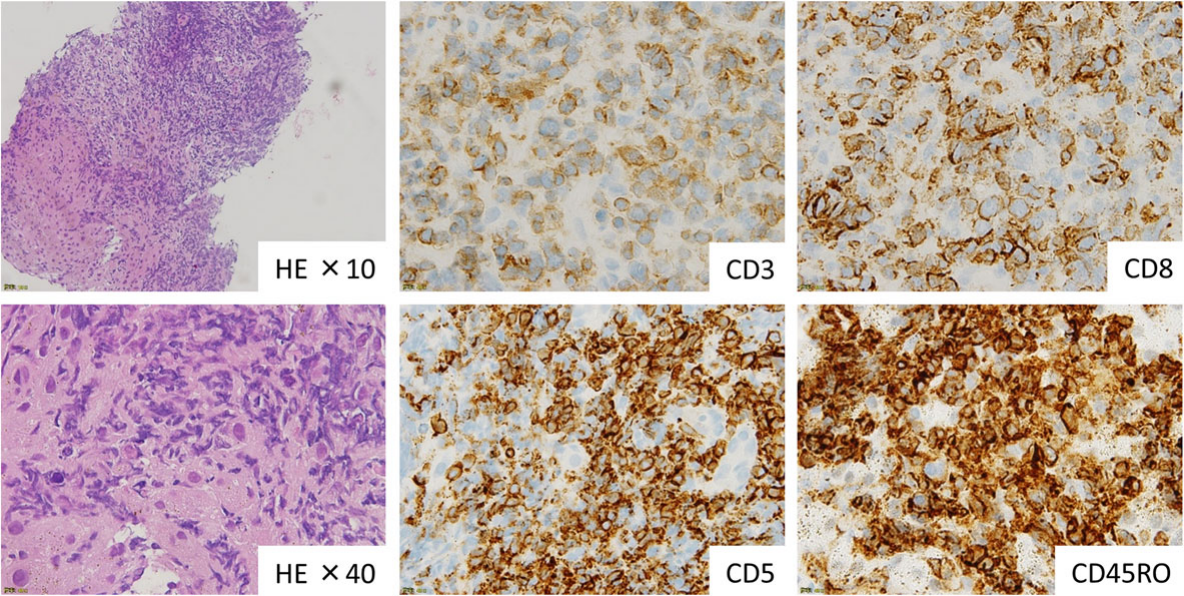
Pathology of peripheral T-cell lymphoma from the liver biopsy. Liver biopsy revealed mid- to large-sized atypical lymphocytes that were phenotypically T cells, positive for CD3, CD5, CD8, and CD45RO with diffuse growth. Lymphoma cells infiltrated the Glisson’s sheath. (CLL; chronic lymphocytic leukemia, DLBCL; diffuse large B-cell lymphoma, PTCL; peripheral T-cell lymphoma)

He underwent ofatumumab therapy, which relieved his febrile lymphadenopathy. He received a total of 11 courses of ofatumumab until disease progression. On the day of the twelfth course of ofatumumab, his body temperature was 38.6 °C and a computed tomography study revealed multiple low-density liver masses that appeared to be similar to multiple liver abscesses. Liver biopsy confirmed T-cell lymphoma of the liver. CD20-lacking CLL cells were confirmed in his peripheral blood and bone marrow by flow cytometry. Moreover, his left cervical pathological diagnosis remained as CD20-positive DLBCL. Thereafter, he was diagnosed with three different types of lymphoma pathologies:

1. CD20-positive DLBCL in the lymph nodes, which was pathologically confirmed in the cervical lymph nodes and transformed from Richter’s CLL (Fig. 1)
2. CD20-lacking CLL in the peripheral blood and bone marrow, which escaped from CD20-positive CLL (Fig. 2)
3. PTCL in the liver (Fig. 3)

He received intravenous and oral dexamethasone therapy as palliative care. He died because of an abdominal emergency due to the rapid progression of multiple liver masses 2 months after the diagnosis of double transformation CLL. Permission was given to perform an autopsy, which confirmed aggressive PTCL systemically involving the liver, spleen, gall bladder, pericardium, bone marrow, and mediastinal–paraaortic–intraceliac lymph nodes. Bone marrow involvement of PTCL was pathologically diffuse and intensive. There was no component of DLBCL in the bone marrow. A T-cell receptor rearrangement study of an autopsy specimen was supportive of a diagnosis of T-cell lymphoma as a synchronous lymphoma (Table 1). A chromosomal study could not obtain active cell division for detecting karyotype in the three types of transformed lymphoma. A cytogenetic study showed that the patient’s original CLL cells harbored del(11)(q?) or its idem, add(15)(q24) clones in all 14 analyzed cells. However, no clonal cytogenetic abnormalities were found in the three types of transformed clones described above (i.e., CD20-positive DLBCL, CD20-lacking CLL, and PTCL cells).

**Table 1:** T-cell rearrangements were identified in the patient’s autopsy specimens by TCR PCR (a) and TCR Southern blotting assay (b)

(a) T-cell receptor rearrangement PCR assay				
TCR chain	Assay region	Cervical	Paraaortic	Liver
TCR γ (TRG)	Vγ1–8,10/Jγ	+	+	+
	Vγ9,11/Jγ	+	–	–
TCR δ (TRD)	Vδ/Dδ/Jδ	–	–	–
TCR β (TRB)	Vβ/Jβ1,2	–	+	+
	Vβ/Jβ2	+	–	–
	Vβ/Dβ1,2	+	–	–
(b) T-cell receptor rearrangement Southern blotting assay				
TCR chain		Cervical	Paraaortic	Liver
TCR Cγ		–	+	+
TCR Cδ1		–	^a^	^a^
TCR Cβ1		–	+	+

^a^Normal rearrangement banding was strikingly weak. The assay suggests gene deletion or other irregularities

## Discussion

Ofatumumab is a humanized monoclonal antibody against CD20 that was designed to bind the CD20 peptide and was initially used for treatment of DLBCL. However, clinical data demonstrated that ofatumumab was active against rituximab-refractory indolent lymphomas [6]. The FDA approved the use of ofatumumab for treatment of CLL refractory to fludarabine and alemtuzumab [5]. Our case was refractory to fludarabine treatment and responded well to salvage treatment with ofatumumab alone. Although evidence for the efficacy of ofatumumab monotherapy against Richter’s syndrome is lacking, some combination regimens of cytotoxic drugs have produced promising outcomes [6, 7]. Although the mechanism of ofatumumab resistance by CLL has not been well characterized [8], in our case, the surfaces of B-CLL cells had no CD20 molecules, suggesting that B-CLL tumor cells lack the CD20 antigen because of transformation during ofatumumab therapy. Some past reports showed the possibility of transformation of CLL to more aggressive lymphoma including T cell lymphoma in concurrently [9] or consequently [10]. In our case, the transformation was followed by ofatumumab therapy and looked being promoted by resistant mechanism.

We observed a surprising replacement of B-CLL by PTCL in our case during autopsy. The clinical course of the PTCL lesion was clearly different from that of CLL/DLBCL. PTCL involved multiple organs and tissues in a highly invasive manner. A past case series reported seven cases of T-cell neoplasm associated with B-CLL [4] with clonal T-cell populations detected in eight of 100 unselected CLL patients and identical clonality of PTCL derived from B-CLL. Through these cases, we speculated that substantial T-cell clonality is present in B-CLL as a result of “clonal drift” beyond B- and T-cell lineages in CLL.

## Conclusion

Richter’s syndrome includes not only transformation to DLBCL but also other types of malignant lymphoid neoplasms, including T-cell lineage neoplasm. This transformation could represent a general potent mechanism in CLL as low-grade B-cell lymphoma.

## Abbreviations

AITL: angioimmunoblastic T-cell lymphoma
CLL: chronic lymphocytic leukemia
DLBCL: diffuse large B-cell lymphoma
FDA: Food and Drug Administration
PTCL: peripheral T-cell lymphoma

## References

[1] Rossi D (2016). Richter's syndrome: novel and promising therapeutic alternatives. Best Pract Res Clin Haematol.

[2] Jain P, O'Brien S (2012). Richter's transformation in chronic lymphocytic leukemia. Oncology (Williston Park).

[3] Jamroziak K, Tadmor T, Robak T, Polliack A (2015). Richter syndrome in chronic lymphocytic leukemia: updates on biology, clinical features and therapy. Leuk Lymphoma.

[4] Martinez A, Pittaluga S, Villamor N, Colomer D, Rozman M, Raffeld M, Montserrat E, Campo E, Jaffe ES (2004). Clonal T-cell populations and increased risk for cytotoxic T-cell lymphomas in B-CLL patients: clinicopathologic observations and molecular analysis. Am J Surg Pathol.

[5] Coiffier B, Lepretre S, Pedersen LM, Gadeberg O, Fredriksen H, van Oers MH, Wooldridge J, Kloczko J, Holowiecki J, Hellmann A, Walewski J, Flensburg M, Petersen J, Safety RT (2008). Efficacy of ofatumumab, a fully human monoclonal anti-CD20 antibody, in patients with relapsed or refractory B-cell chronic lymphocytic leukemia: a phase 1-2 study. Blood.

[6] Cheson BD (2010). Ofatumumab, a novel anti-CD20 monoclonal antibody for the treatment of B-cell malignancies. J Clin Oncol.

[7] Matasar MJ, Czuczman MS, Rodriguez MA, Fennessy M, Shea TC, Spitzer G, Lossos IS, Kharfan-Dabaja MA, Joyce R, Fayad L, Henkel K, Liao Q, Edvardsen K, Jewell RC, Fecteau D, Singh RP, Lisby S, Moskowitz CH (2013). Ofatumumab in combination with ICE or DHAP chemotherapy in relapsed or refractory intermediate grade B-cell lymphoma. Blood.

[8] Baig NA, Taylor RP, Lindorfer MA, Church AK, LaPlant BR, Pettinger AM, Shanafelt TD, Nowakowski GS, Zent CS (2014). Induced resistance to ofatumumab-mediated cell clearance mechanisms, including complement-dependent cytotoxicity, in chronic lymphocytic leukemia. J Immunol.

[9] Strickler JG, Amsden TW, Kurtin PJ (1992). Small B-cell lymphoid neoplasms with coexisting T-cell lymphomas. Am J Clin Pathol.

[10] Lee A, Skelly ME, Kingma DW, Medeiros LJ (1995). B-cell chronic lymphocytic leukemia followed by high grade T-cell lymphoma. An unusual variant of Richter's syndrome. Am J Clin Pathol.

